# Environmental preferences and critical habitat for the velvet belly lanternshark (*Etmopterus spinax*) in Icelandic waters

**DOI:** 10.1371/journal.pone.0299544

**Published:** 2024-03-13

**Authors:** Helga Mattína, Steven E. Campana, Klara Jakobsdóttir

**Affiliations:** 1 Life and Environmental Science, University of Iceland, Reykjavík, Iceland; 2 Marine and Freshwater Research Institute, Hafnarfjörður, Iceland; Instituto Portugues do Mar e da Atmosfera, PORTUGAL

## Abstract

The velvet belly lanternshark (*Etmopterus spinax*) is a small, bioluminescent shark that is caught as bycatch in many deep-sea fisheries in the Atlantic Ocean. Using data from 10,597 seasonal research survey tows spanning 11 years, the distribution, relative abundance, life history, and environmental preferences of *E*. *spinax* in Icelandic waters was examined for the first time. *E*. *spinax* (n = 8774) were only captured in relatively deep offshore waters to the south and west of Iceland. Females grew to larger sizes than males and reached 50% sexual maturity at a total length of 50 cm. Females at a late stage of maturity and very small juveniles (<20 cm) were restricted to the central south Icelandic shelf, suggesting that this might be critical habitat for the reproduction of the species. Most of the sharks were captured at depths of 400–500 m, a relatively narrow depth range, and classified as a stenothermic warm-water species with habitat temperature restricted to about 6.3–8.0°C. Teleosts, crustaceans and cephalopods made up most of the diet. There was no indication of a decline in abundance over the time span of the survey. However, climate-induced warming of the deep ocean may shift the distribution of the species to more northerly waters within Iceland.

## Introduction

The life history of sharks is characterized by slow growth rates, late sexual maturity, low fecundity and long lifespans [1]. This makes them exceptionally vulnerable to exploitation, as low productivity rates make them less able to recover from overfishing [2,3]. Overfishing poses a serious threat of extinction to one third of all shark species and other chondrichthyans [4]. In particular, deep-sea sharks are believed to be vulnerable to potential overfishing, as they appear to have low rates of population rebound leading to high extinction risks, although they are considered to be less vulnerable than pelagic and coastal sharks due to their depth refuge [2,5]. However, very little is known about the life history and distribution of most deep-sea sharks [5,6]. Many deep-sea sharks are of little to no commercial value, yet can be commonly caught as bycatch in deep-water fisheries [6]. Official landing and bycatch numbers for these species are seldom reported [3].

The velvet belly lanternshark, *Etmopterus spinax* (Linnaeus, 1758) belongs to the genus *Etmopterus* spp. (family Squalidae) [7] which includes approximately 52 species [8]. Lanternsharks are small, usually smaller than 80 cm [9]. In the Atlantic Ocean the distribution of *E*. *spinax* ranges from the north Atlantic Ocean, off the shores of Iceland and Norway, down along the western coast of Africa, extending into the western Mediterranean Sea. It can be found at depths ranging from 70 to 2000 meters but is most commonly found at depths between 200 to 500 meters [7]. The species is sexually dimorphic, with the females growing larger than the males [10–13].

*Etmopterus spinax* is commonly caught as by-catch in many deep-sea fisheries [10–14]. The species is of little or no commercial value and is usually discarded [10–13,15]. For that reason, little effort has been put into research on this species in Icelandic waters. The species is listed as “Vulnerable” by the IUCN Red List [16].

Although some aspects of the life history and feeding habits of *E*. *spinax* have been reported for the northeastern Atlantic Ocean [10–12] and the Mediterranean Sea [13,14], little is known about the species in Icelandic waters other than its spatial distribution [17,18]. Using data from the Marine and Freshwater Research Institute (MFRI) annual spring and autumn research surveys, the objectives of the current research were to enhance our knowledge of the life history, sexual maturity and environmental preferences of *E*. *spinax* in Icelandic waters, as well as analyze its long-term trends of distribution and abundance. The study concludes with the implications of these results for the conservation of the species in light of ongoing fishery practices and climate change.

## Methods

**Fig 1:**
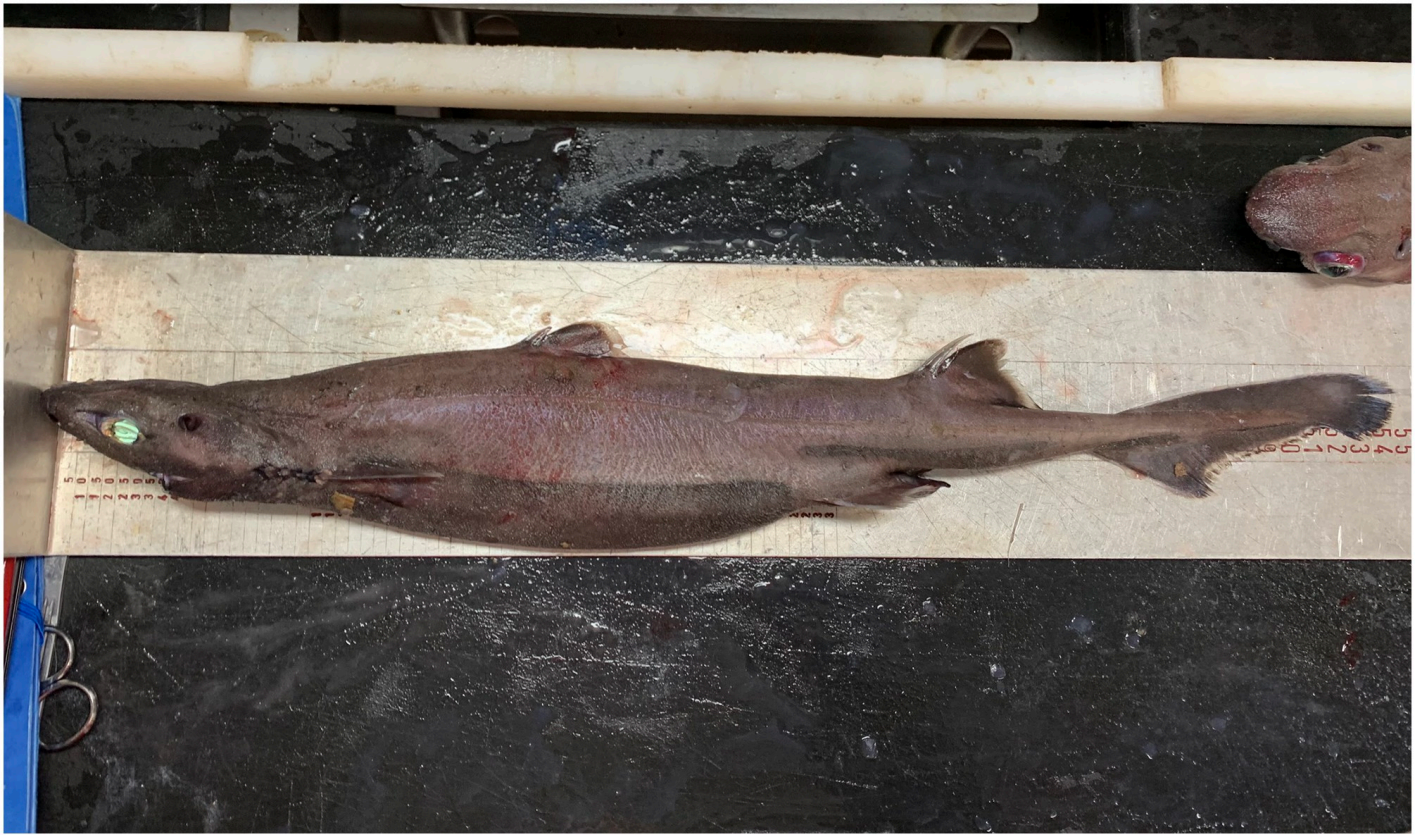
A female Etmopterus spinax (54 cm TL) caught during the autumn research survey in October 2021.

Samples of *Etmopterus spinax* (Fig 1) were collected by the Marine and Freshwater Research Institute (MFRI) of Iceland during spring and autumn bottom trawl research surveys conducted biannually from 2010–2021.

AAll *E*. *spinax* were counted at each station; subsamples were taken for total length (TL to the nearest cm), weight (to the nearest g), sex and maturity, following MFRI protocols [19].

During the autumn research surveys a maximum of 20 individuals were sexed and measured at each station. A maximum of five individuals were weighed and examined for maturity stage. During the spring survey, at least one *E*. *spinax* was sexed and measured, but maturity stage was not recorded. Sex was determined by the presence or absence of claspers, while maturity was assessed based on the examination of the reproductive organs using the Stehmann [20] maturity stage scale. Under this scale, female maturity was scored from 1 to 7, with stage 1 classed as immature, stages 2 and 3 have developing oocytes, stage 4 has fertilized eggs in the uterus, stages 5 to 6 have developing embryos, and stage 7 is spent.

Sharks ≤ 20 cm were assumed to be juvenile and their capture locations were assumed to best represent any potential nursery area.

Maturity ogives were estimated by fitting a logistic regression to the sex-specific length-maturity data using the R package SizeMat v1.1.2 [21].

Any given fish species tends to have a specific temperature range with which it is most strongly associated. The thermal bias of an individual species may be towards waters that are warmer or colder than that of the overall environment (i.e. a warmwater or coldwater species). The thermal bias (TB) of *E*. *spinax* was calculated as the median bottom water temperature of all stations across all years subtracted from the median catch-weighted temperature of the shark across all years [22]. Thus a positive TB index would be indicative of a warmwater species, while negative TB indices would be classified as coldwater. In addition, the range of temperatures which can be tolerated or preferred can either be narrow (stenothermal) or broad (eurythermal). To characterize the temperature tolerance range, the Steno index was calculated as the temperature range delimited by the 5th and 95th percentiles of the species’ catch-weighted temperatures across all years [22]. Thus a small value for the Steno index would be indicative of a species with a very narrow temperature tolerance range.

Cumulative distribution functions (CDFs) were used to compare the temperatures where *E*. *spinax* were caught compared with all stations which were fished. The unweighted CDF represents the temperatures at all stations, while the weighted CDF indicates the temperatures associated with the same series of fishing sets, weighted by the catch rate of the shark. The range of temperatures encompassed by the 10th and 90th percentiles indicates the range of temperatures where most (80%) of the fishing activity and/or catches took place. Similarities between weighted and unweighted CDFs suggest that enhanced catches are not associated with any particular range of temperatures within the range fished, while different CDFs suggest temperature associations. CDFs were used in the same manner to examine the depth associations of *E*. *spinax*.

Although not the primary focus of the study, additional samples were collected for stomach content analysis (n = 27) and female egg counts while onboard Árni Friðriksson HF-200 in early October 2021 as part of the annual MFRI autumn research survey. Stomach sampling stations were located along the southwest Iceland continental shelf at depths ranging from 68 to 1342 m. Stomach contents were weighed to the nearest gram and assigned visually to one of six categories: empty, teleost, crustaceans, cephalopods, echinoderms or unidentified.

All fish samples were collected on annual Icelandic federal government research surveys with the approval of the Animal Care Committee of the Marine and Freshwater Research Institute in Reykjavik.

The data were analyzed using R statistical software v4.1.1 [23]. Distribution was mapped with the maps R package v3.4.0 [24]. The weight-length relationship was analyzed using the FSA R package v0.9.1 [25]. Maturity ogives were plotted using the R package sizeMat v1.1.2 [21]. Additional statistical analysis and plotting were created using the R package tidyverse v1.3.1 [26].

## Results

A total of 10,597 tows spanning 11 years were examined for this study. Through this period, stations were sampled at bottom depths ranging from 18 to 550 m during the spring research surveys, and 23 to 1359 m during the autumn surveys. Bottom temperatures ranged from -0.8 to 8.2°C during the spring research surveys, and -1.0 to 11.0°C during the autumn surveys. Of the 8774 *E*. *spinax* that were caught in both seasonal surveys, 54% were measured, 12% were weighed, 50% were sexed and 12% were staged for maturity.

**Fig 2:**
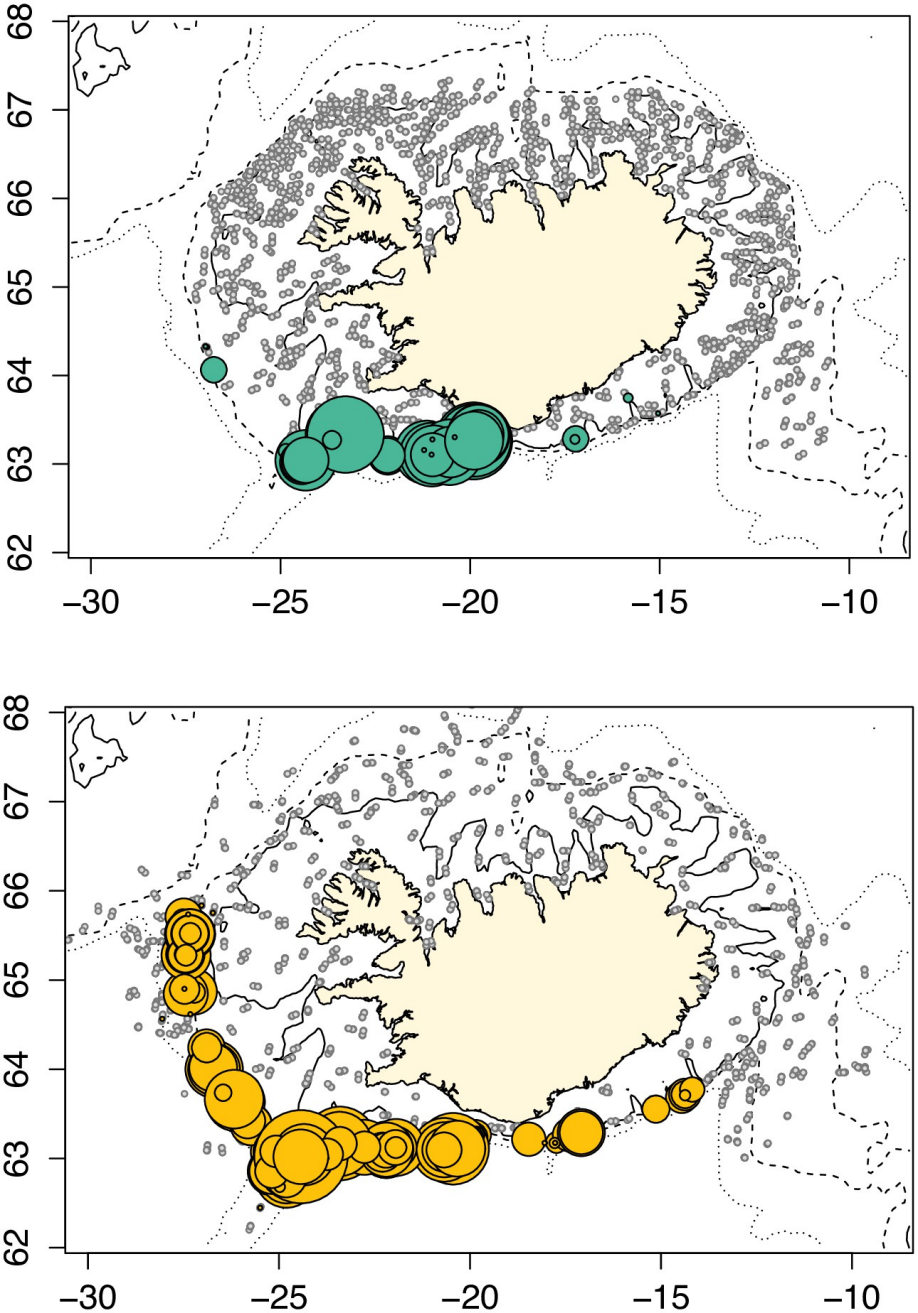
Seasonal variation in distribution of E. spinax caught during the 2010–2021 MFRI spring (green) and autumn (orange) research surveys, whereby the size of the symbol is proportional to the number of E. spinax in the catch. Grey circles represent null catches. A total of 3950 sharks were caught in the spring survey, and 4614 sharks in the autumn survey.

Survey catches of *E*. *spinax* were restricted to the southern and western regions of Iceland; none were caught in the north or east (Fig 2). The distribution varied considerably between the spring and autumn research surveys with a much broader distribution in the autumn (n = 4614 sharks) than in the spring (n = 3959 sharks).

**Fig 3:**
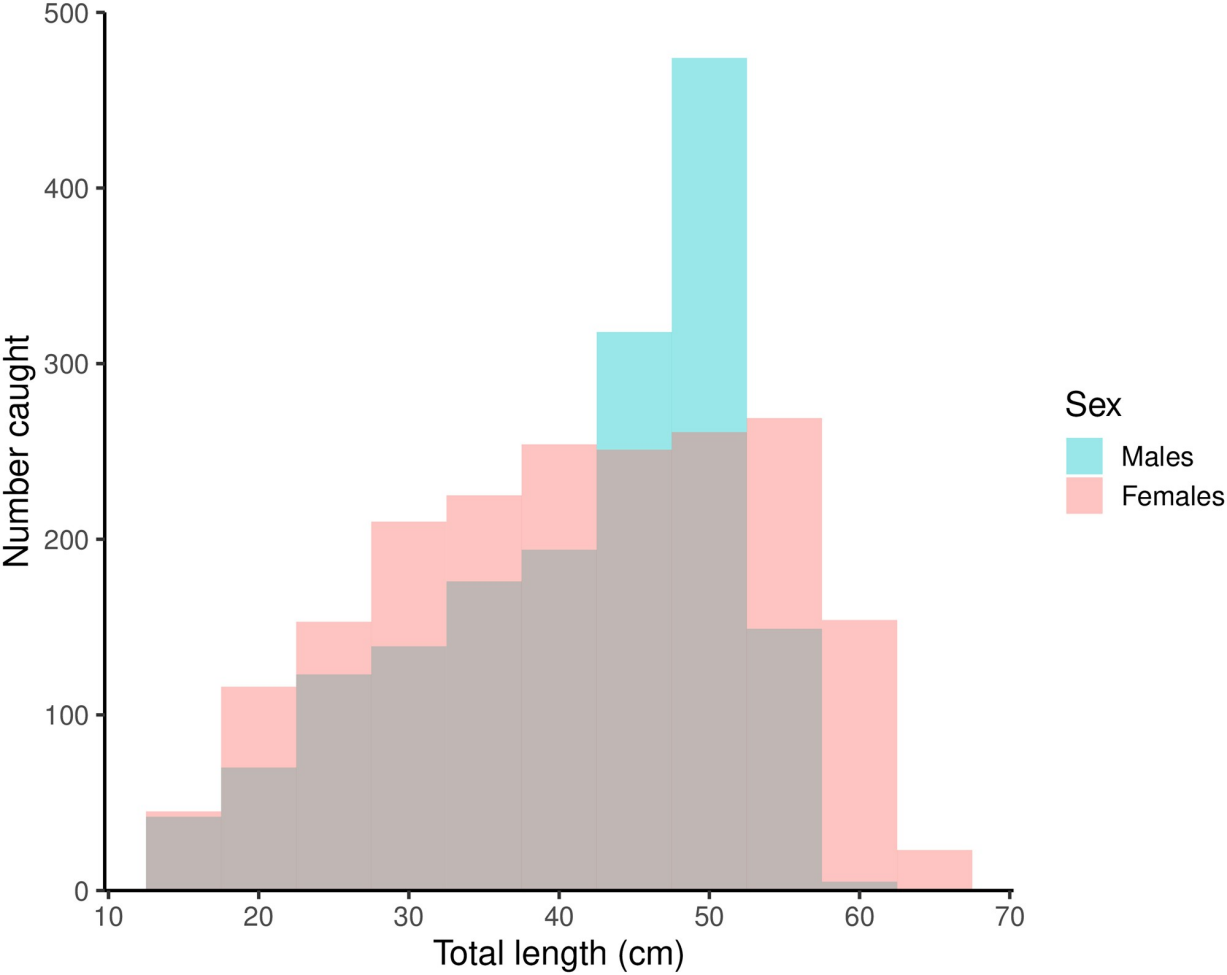
A histogram showing the differences in length distribution of caught E. spinax for males (blue) and females (red).

The overall size range of the *E*. *spinax* that were caught was 13 to 66 cm TL; however females reached larger maximumsizes than the males (Fig 3). Males ranged from 13 to 62 cm (mean = 41 cm) with weights of 11 to 950 g (mean = 401 g) while female lengths ranged from 13 to 66 cm (mean = 41 cm) with weights of 16 to 1606 g (mean = 473 g). Among individuals assessed for maturity stage, immature females ranged in length from 16 to 64 cm (mean = 39 cm) and weighed from 16 to 1284 g (mean = 302 g), while the size of mature females ranged from 28 to 66 cm (mean = 54 cm) and weighed from 25 to 1606 g (mean = 733 g). Immature males ranged in length from 14 to 58 cm (mean = 36 cm) and their weight ranged from 11 to 723 g (mean = 225 g), while mature males ranged in size from 38 to 57 cm (mean = 50 cm) and weighed from 223 to 950 g (mean = 513 g). The pooled-sex length-weight relationship was well described by the equation:

W=−5.73xTL^3.06,P<0.005;r2=0.98

**Fig 4:**
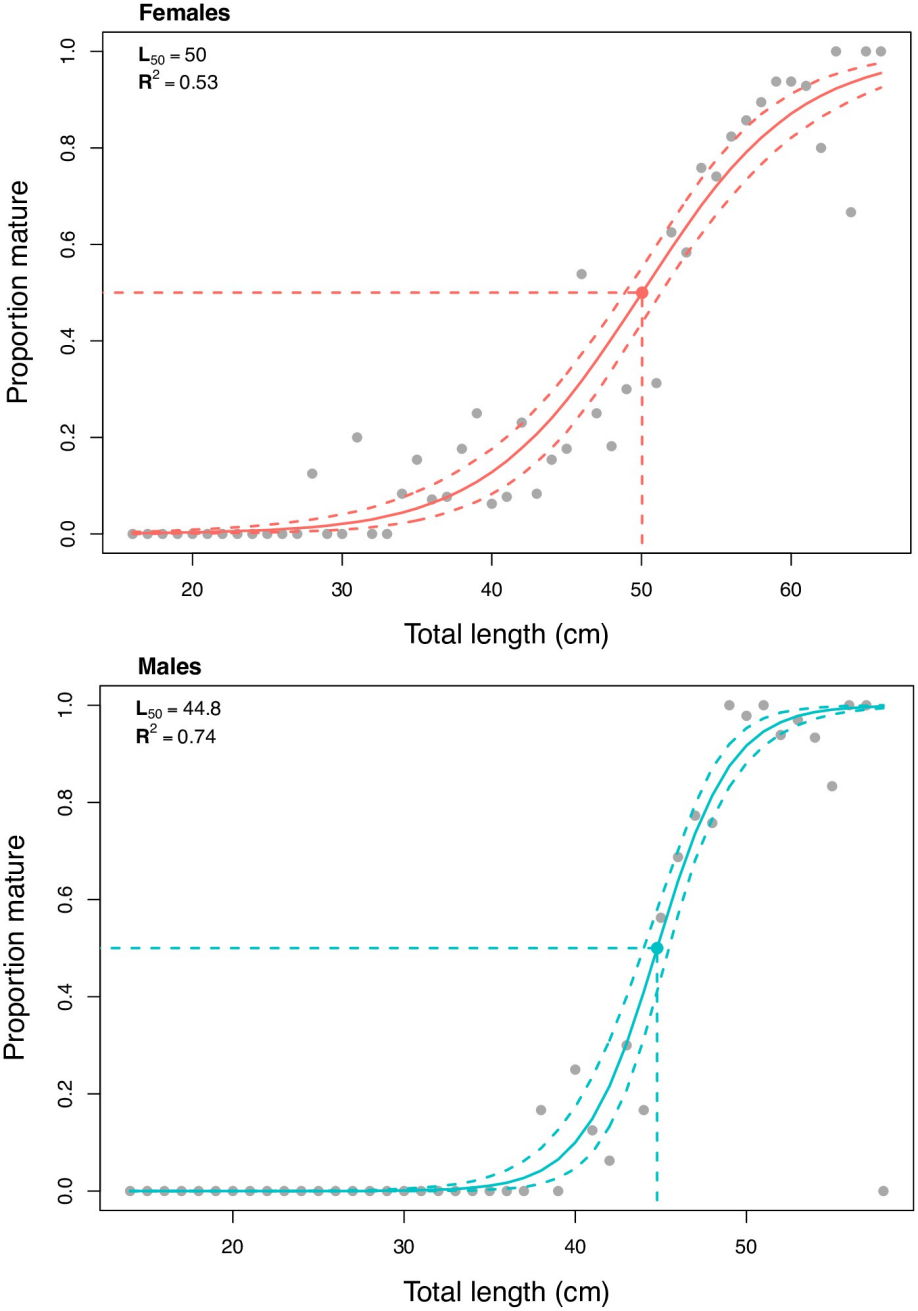
Length-based maturity ogive of E. spinax sampled (n = 1078) during the 2010–2021 research surveys, showing the proportion mature at a given length. The female maturity ogive (n = 582) (red) on top and the male maturity ogive (n = 496) (blue) below.

The maturity ogive showed that female *E*. *spinax* reach maturity at larger sizes than the males (Fig 4), with a female length at 50% maturity of 50.0 cm (TL) (76% of the maximum observed size). Full female maturity appeared to have been reached at lengths greater than 60 cm. The length at 50% maturity for males was 44.8 cm (TL) (77% of the maximum observed size), with full maturity reached at lengths greater than 48 cm.

Out of the 14 females that were staged for maturity in October 2021, six had ripe oocytes. The number of oocytes ranged from 19 to 27, with an overall mean of 11.5. The relationship between number of eggs and total weight was not significant (ANOVA, P = 0.067, r^2^ = 0.51).

**Fig 5:**
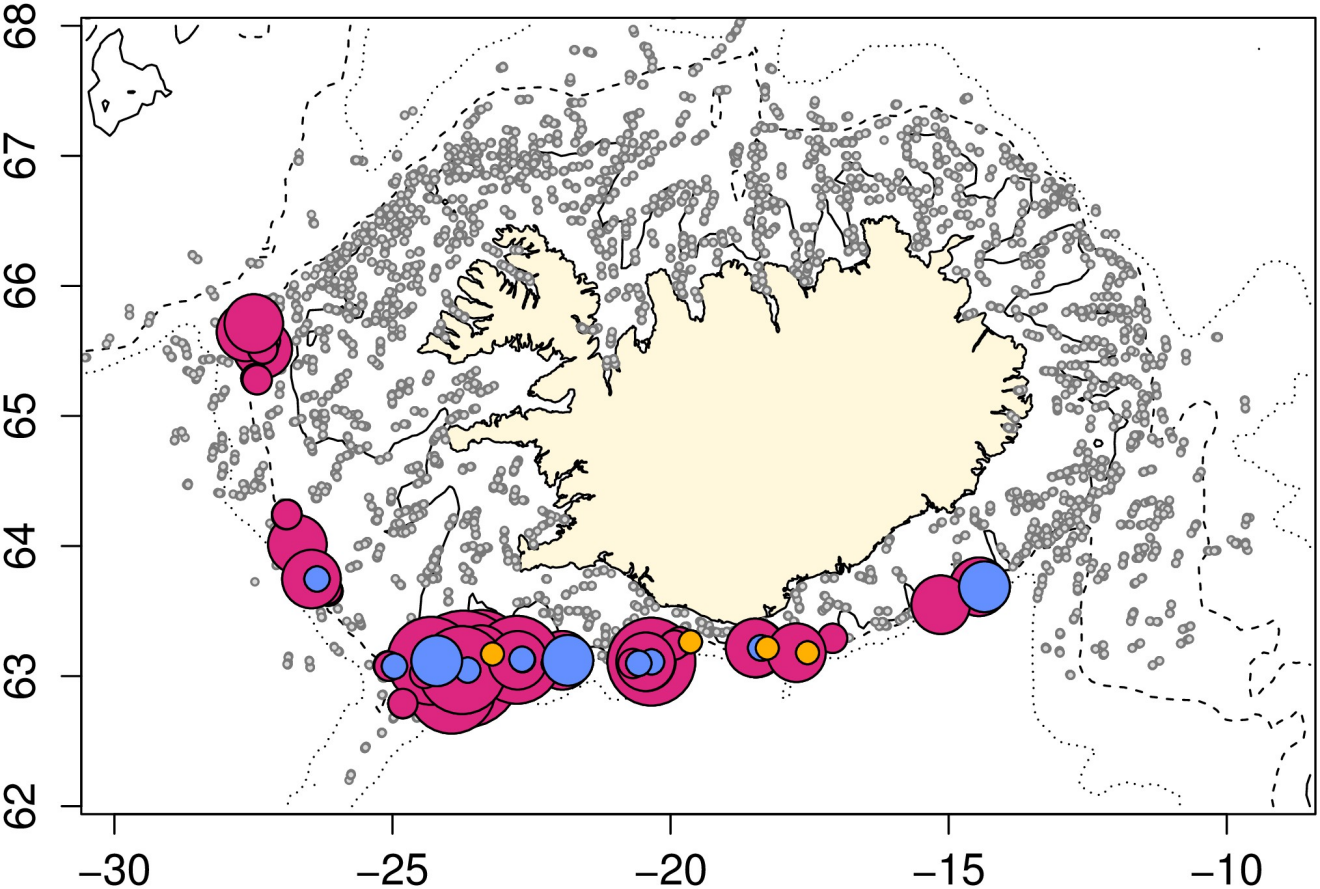
Distribution of mature female E. spinax caught during the 2010–2021 autumn research surveys. Maturity stage 5 (n = 17) shown in blue, maturity stage 6 (n = 4) shown in orange, all mature stages (3–7; n = 111) shown in magenta, stations with no mature females shown in grey. Maturity stages 5 and 6 are both pregnant females, where stage 5 has small embryos and stage 6 has fully developed embryos.

Males (n = 2107) and females (n = 2278) were caught throughout the distribution range of *E*. *spinax*, but more females were caught in the southern portion of the range. Mature females (n = 111) were caught throughout the distributional range (Fig 5) at depths ranging from 191 to 686 m (mean = 481 m). Maturity stages 5, 6 and 7, which are the best indicators of imminent reproduction, were restricted to the southern shelf region of Iceland. In particular, stage 6 (which has fully developed embryos) were found only in the south central portion of the Icelandic shelf.

**Fig 6:**
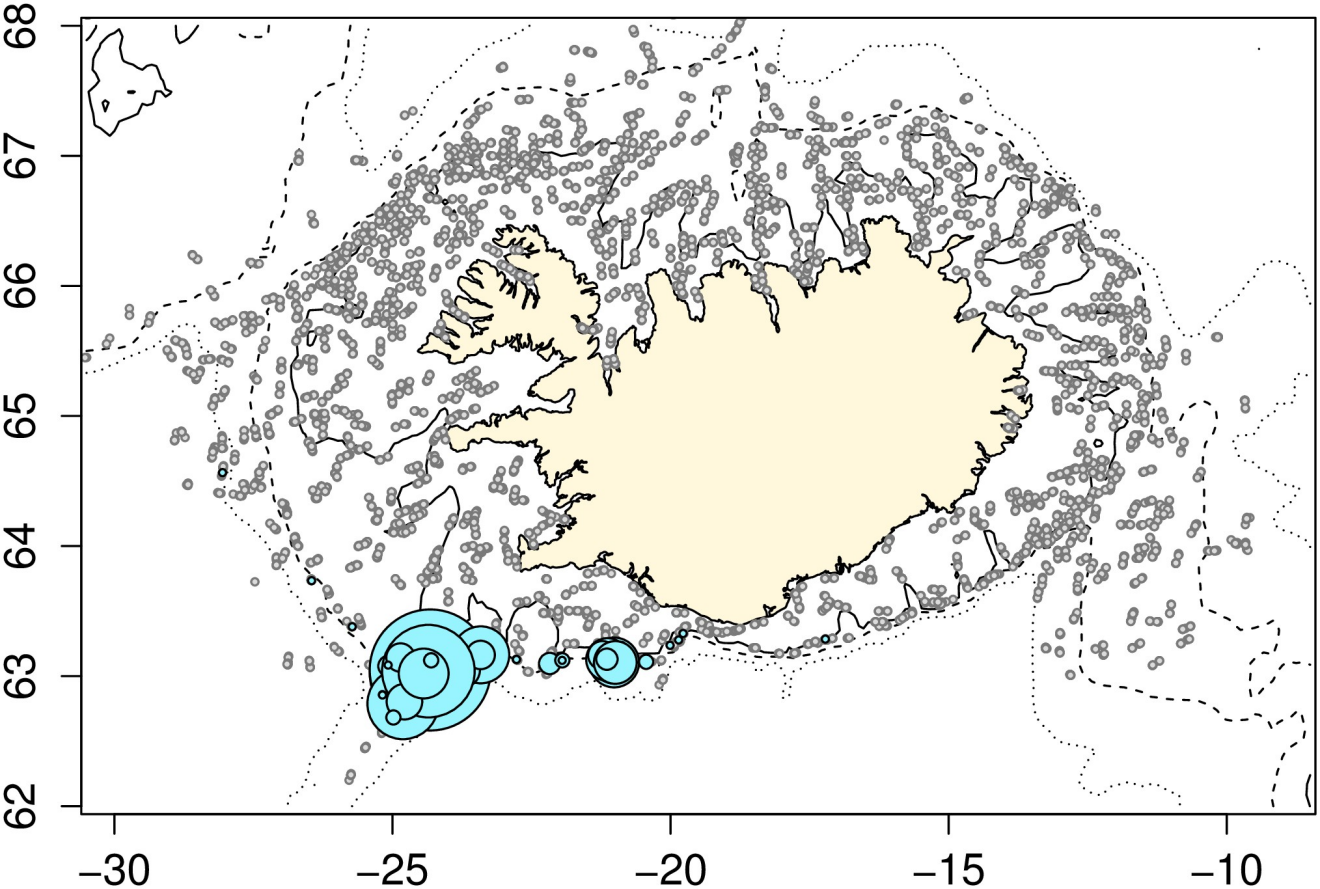
Distribution of juvenile E. spinax (measuring ≤ 20 cm in total length; n = 298) caught during the 2010–2021 MFRI research surveys. Expanding symbol sizes are proportional to catch numbers.

The distribution of juveniles (maturity stage 1) was similar to the overall distributional range of the species. Stage 1 juveniles (n = 537) were caught at depths ranging from 178 to 1049 m (mean = 436 m). Individuals measuring ≤ 20 cm in total length (n = 298) were restricted to the southwestern region of the survey area, which covers the Reykjanes ridge and its surroundings, (Fig 6) at depths ranging from 141 to 995 m (mean = 406 m).

**Fig 7:**
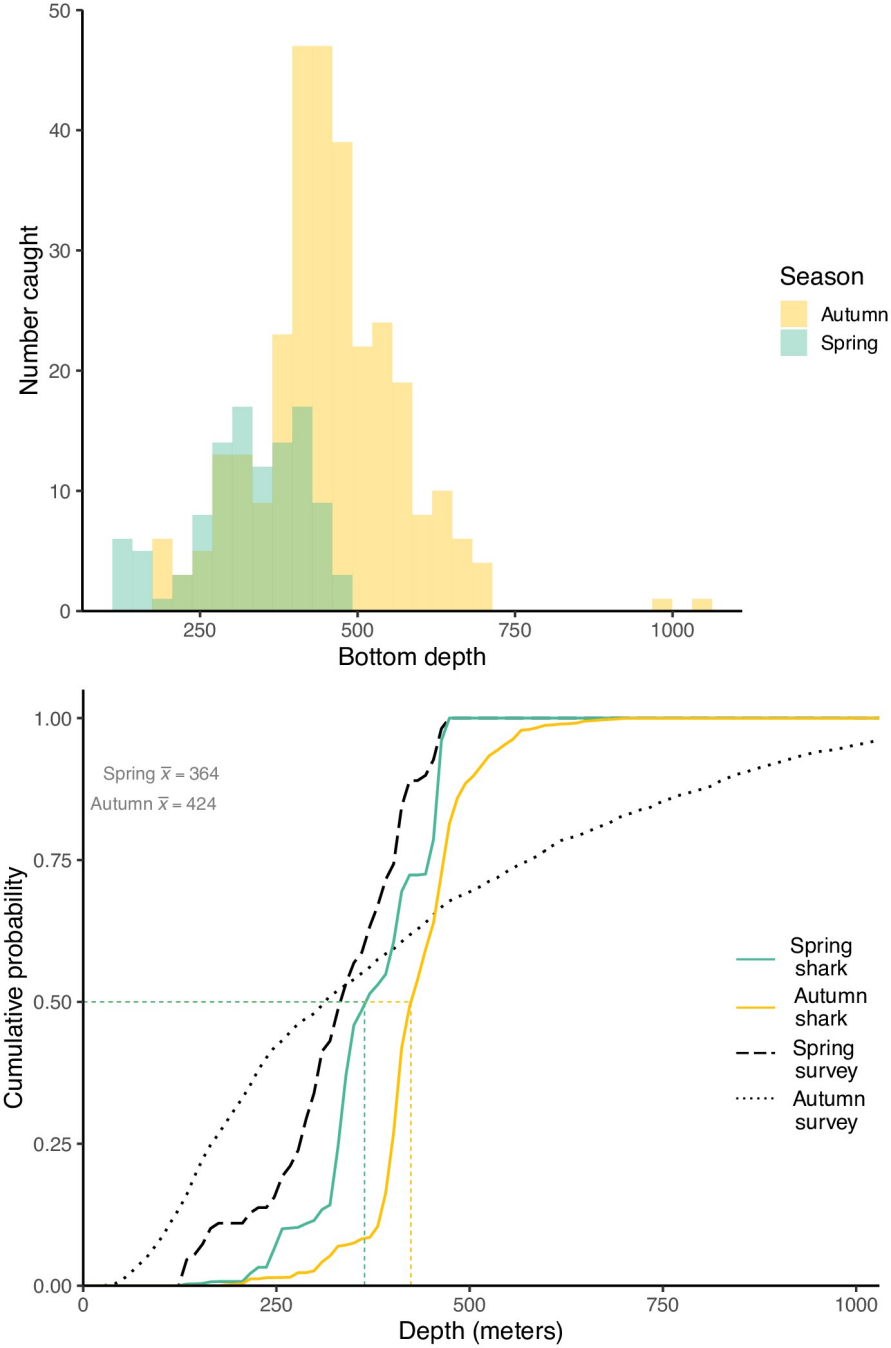
Histogram (top) and cumulative distribution function (CDF) (bottom) of tow depth during the 2010–2021 MFRI research surveys. Stations where E. spinax was caught during the spring and autumn research surveys are shown in orange and green, respectively. The black dashed line shows the depth CDF for all stations in spring, while the dotted black line shows the depth for all stations in the autumn.

*Etmopterus spinax* of all sizes were caught at survey depths of 129 to 1049 m (mean = 416 m), (Fig 7). Cumulative distribution functions (CDFs) demonstrated that *E*. *spinax* were captured in a relatively narrow range of depths compared to those sampled by all stations. For example, while station depths in the autumn ranged from 23 to 1359 m, 90% of *E*. *spinax* were captured at depths of 315 to 546 m. The 90% CDF range for sharks captured in the spring was 241 to 461 m.

**Fig 8:**
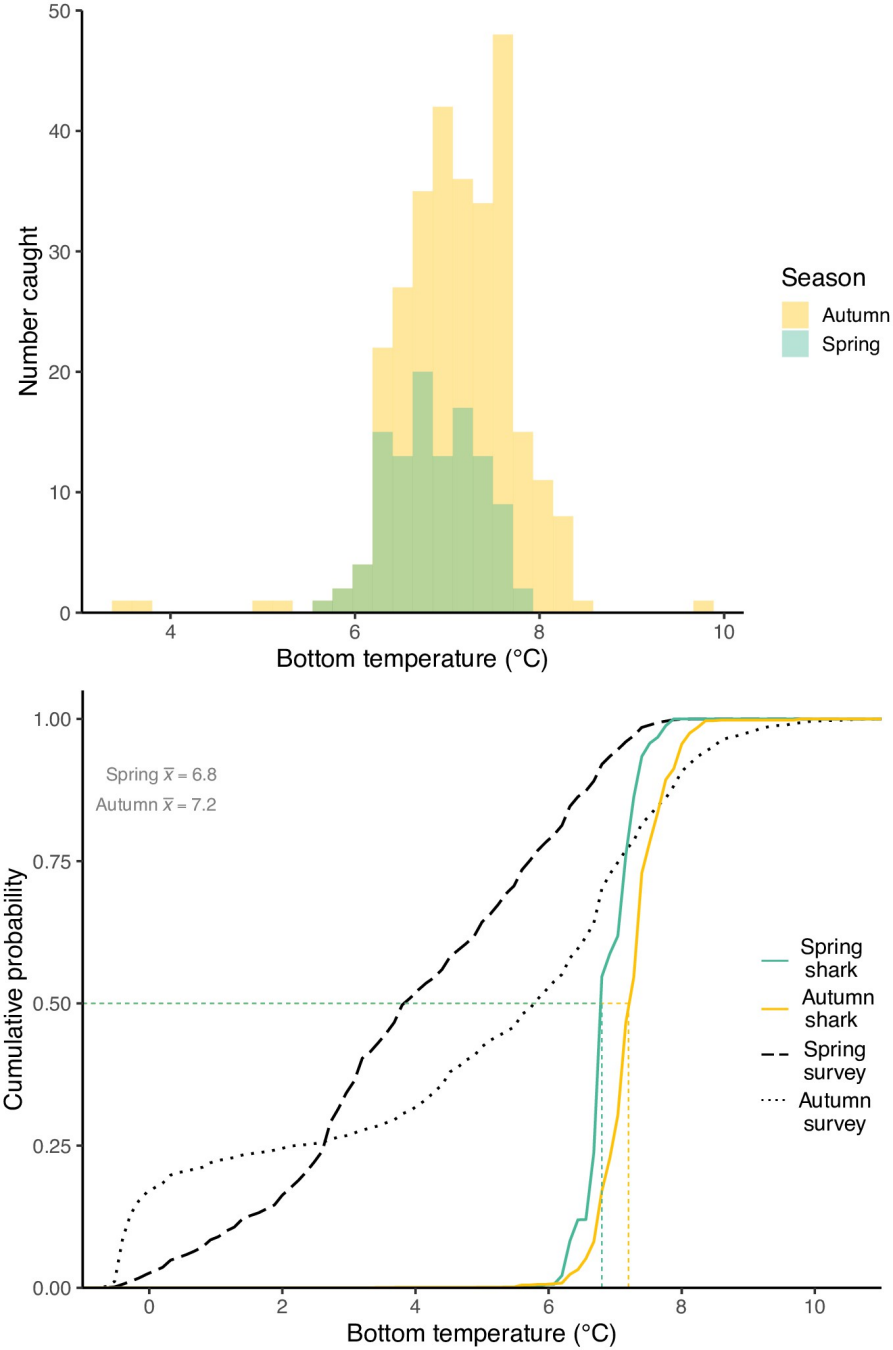
Histogram (top) and CDF (bottom) of bottom temperature during the 2010–2021 MFRI research surveys. Stations where E. spinax was caught during the spring and autumn research surveys are shown in green and orange, respectively. The black dashed line shows the bottom temperature CDF for all stations in spring, while the dotted black line shows the bottom temperature for all stations in the autumn.

*Etmopterus spinax* were caught at survey temperatures of 3.4 to 9.7°C (mean = 7.1°C), but the temperature differed significantly between the spring and autumn surveys (ANOVA, P < 0.01) (Fig 8). The temperature distribution in the spring (range of 5.7 to 7.8°C, mean = 6.9°C) was significantly lower than in the autumn (range of 3.4 to 9.7°C, mean = 7.1°C). It is possible that the ambient temperature difference between the seasons was due to the restricted station depth coverage in the spring. Cumulative distribution functions (CDFs) demonstrated that *E*. *spinax* were captured in a very restricted range of temperatures compared to the overall temperature of all stations (Fig 8). For example, station temperatures in the autumn ranged from -1 to 11°C, but *E*. *spinax* were only captured at temperatures of 3.4 to 9.7°C, with 90% found between temperatures of 6.5 to 8.0°C. The 90% CDF range for sharks captured in the spring was 6.3 to 7.5°C compared to a 90% range of 0.4 to 7.1°C for the stations.

The stenothermal index of *E*. *spinax* was 2.09 (90% of the sharks were concentrated within a 2.09°C temperature range), indicating that the species occupied a very narrow temperature range and thus was very stenothermal. The thermal bias index for the species was 1.46 (the mean temperature of the stations where sharks were caught was 1.46°C warmer than that of the mean of all stations), which classifies it as a warmwater species.

Out of the 27 stomachs examined, 19% were empty and 81% contained food items. Teleost remains were the most common item found in 50% of the stomachs, followed by crustaceans (41%), cephalopods (18%), unidentified (14%), and echinoderms (5%). Teleost remains accounted for 82% by weight while crustaceans accounted for 11%, cephalopods 7%, unidentified 7% and echinoderms 3% by weight.

**Fig 9:**
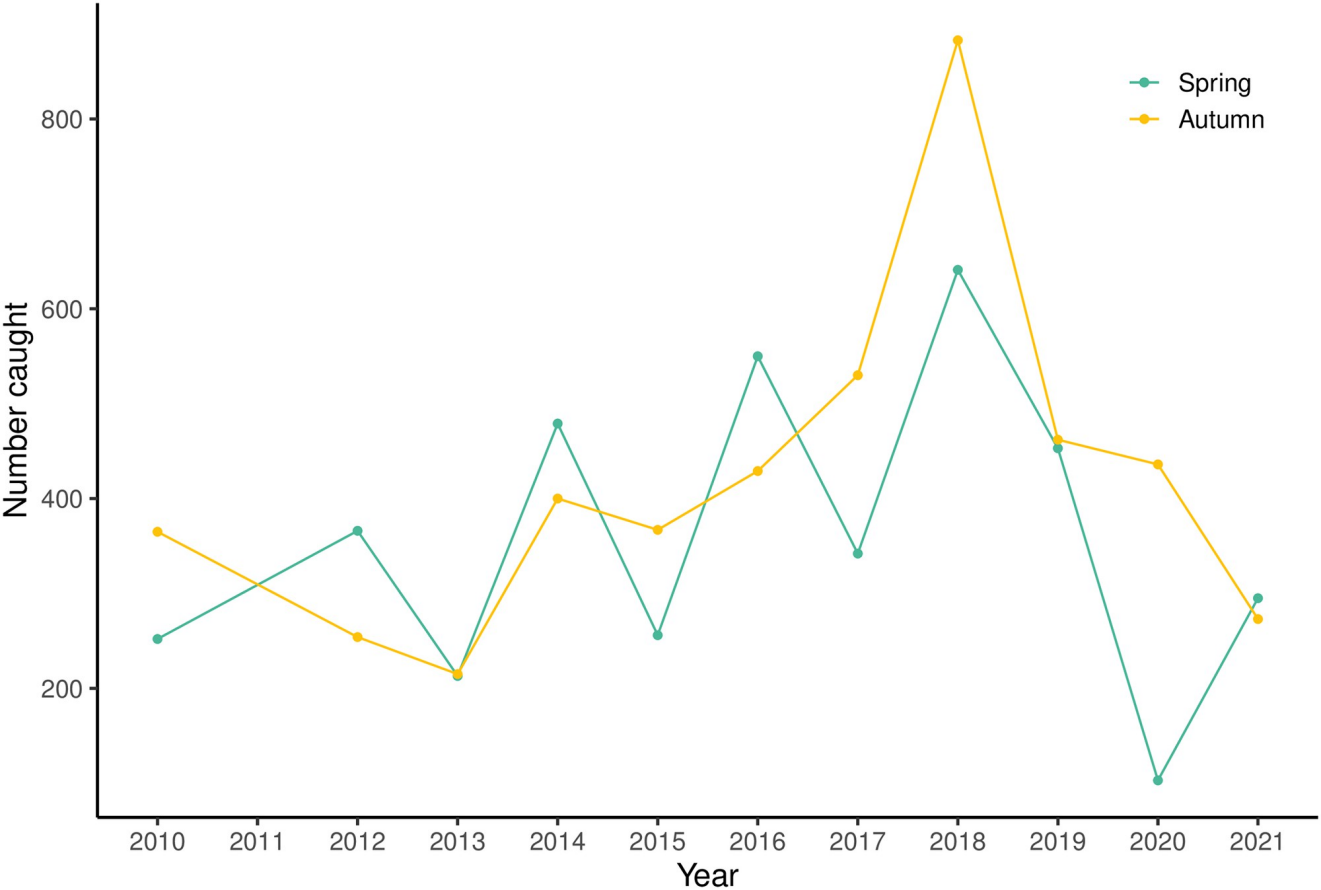
Annual catch number index of E. spinax (n = 8774) during the 2010–2021 MFRI spring and autumn research surveys.

There was no change in net relative abundance of *E*. *spinax* across the 11 year timespan of the research surveys (Fig 9). Both the spring and autumn surveys were relatively synchronous, showing an increasing trend in net relative abundance between 2010 and 2018, with some decline in the last two years.

## Discussion

Exploitation of deep-sea habitats poses an increasing risk to vulnerable non-commercial bycatch species, such as deep-sea sharks [2,6]. However, conservation risk can only be evaluated when the productivity and distribution of the species is known [27,28]. This study provides baseline information on the distribution of *E*. *spinax* in Icelandic waters as well as on its habitat preferences, and is one of only a handful of studies on the species worldwide. The ecology, population dynamics and life history of *E*. *spinax* is poorly known, as is the case for many other deep-sea sharks around the world [5,6].

The distribution range of *E*. *spinax* caught during 11 years of research surveys was restricted to the warmer waters south and west of Iceland [22,29] ö. The vast majority of all *E*. *spinax* sampled in this research project were caught at bottom temperatures ranging from 6 to 8°C, which contrasts to the previously observed temperature range reported by Jac *et al*. [18] of 4 to 5°C. Although both studies are based on the same surveys, the analysis reported here covers a longer period of time (11 yr; 2010–2021), than the three years (2018–2020) analyzed by Jac *et al*. [18]. Although Jakobsdóttir [30] reported depth segregation by sex and size for the deep-sea sharks *Centroscyllium fabricii* and *E*. *princeps* in Icelandic waters, we did not see a similar depth segregation with *E*. *spinax*.

*Etmopterus spinax* in Icelandic waters are substantially larger than any previously sampled in the northeastern Atlantic Ocean and in the Mediterranean. In the northeastern Atlantic Ocean around Portugal, the TL has been observed to range from 9.1 to 41.1 cm for females and 10.2 to 33.8 cm for males [10–12]. In the central western Mediterranean Sea, Porcu *et al*. [13] reported the TL for females to be 10.0 to 45.7 cm and 9.7 to 41.8 cm for males. The largest individual sampled during our research surveys was a 66.0 cm long female, which is the largest confirmed maximum total length reported for the species.

Female *E*. *spinax* in Icelandic waters reached maturity at a greater length than the males, a commonly observed pattern for *E*. *spinax* in other areas [10–13] and for squaloid sharks in general [30–32]. However, the length at maturity of 50 cm appears to be larger in Icelandic waters than in other areas. In other areas of the northeastern Atlantic Ocean, *E*. *spinax* female length at 50% maturity has been reported to vary between 30.9 to 36.9 cm (TL) [13,33]. As a percentage of the maximum observed size though, all areas seem to be similar at around 75% for females and 77% for males.

It has been observed in the Atlantic Ocean that more northern populations reach larger maximum sizes and longer lengths at 50% maturity than southern populations [34]. Colder temperatures have been shown to cause slower growth rates but larger maximum size [35,36], and within species, larger individuals have been found to prefer colder waters [37]. Higher fishing mortality rates are also known to cause populations to reach maturity at smaller sizes and have smaller maximum sizes [38,39].

Age at sexual maturity was not estimated in this study. On the basis of vertebral band counts, Gennari and Scacco [14] estimated the maximum age of *E*. *spinax* caught in the Tyrrhenian Sea to be 9 and 7 years for females and males, respectively. On the basis of band counts on the second dorsal spine, Coelho and Erzini [10] reported maximum ages of 11 and 8 years for females and males, respectively. In both studies however, the aging method was of unknown accuracy. Age determination of deep-sea sharks using dorsal spines can be problematic due to poorly visible banding [40]. Shark ages determined using methods of known accuracy have yielded much greater ages than previously believed for almost all shark species [31,40,41], making it likely that age at maturity has been underestimated for *E*. *spinax*.

*Etmopterus spinax* was most abundant at depths of around 400 to 500 m, somewhat deeper than the depths of 300 to 400 m reported in a preliminary study in Icelandic waters [18]. Although caught at depths spanning over 900 m, the great majority of sharks were captured within a 220 m range in the spring and a 231 m range in the autumn, indicating that the species prefers a narrow depth range. These depths are much shallower than have been reported for the species in other areas. In the waters off Portugal the species was most commonly caught from 500–599 m [11,42], while in the Mediterranean it was most commonly caught at depths over 700 m [42], but with a seemingly wider depth range. The temperature CDFs indicated that *E*. *spinax* are extremely stenothermic. Although the median temperature of the spring shark habitat was significantly different than that of the fall, the absolute magnitude of the difference was only 0.2°C. Thus it appears likely that some or most of the shift in seasonal depth distributions can be attributed to temperature preferences. Similar temperature-driven movements have been reported for other deep-sea species [43,44]. Over the long term, climate-driven shifts in distribution might be expected to occur, as has been observed for many other sub-Arctic fish species [22]. Since our results indicate that *E*. *spinax* can be classified as a warm-water species, and given its current distribution in the warm southern waters of Iceland, then any climate-driven shifts in location might be expected to be a range expansion towards more northerly waters.

The diet of *E*. *spinax* in Icelandic waters consists largely of teleost, crustaceans, and cephalopods, which is comparable to feeding habits observed in other areas [12,45], although the specifics vary from region to region [46–48]. As the diet of E. spinax was only a small component of this study, future research should aim to look into the specific feeding habits of the species in Icelandic waters. Critical habitat for marine species is usually defined as areas required for spawning, nursery areas and feeding [49]. As a deep-sea species, feeding habitat for *E*. *spinax* is presumably widespread. However, our results indicate that spawning (birthing) habitat is much more restricted in distribution. The distribution of mature females in the final stage of embryo release was restricted to the warm, southerly waters of Iceland, an area which overlapped the restricted distribution of very small (≤20 cm) juveniles, although it was not clear if any of the juvenile sharks had been born immediately prior to capture. Thus this region of the offshore, at depths and temperatures of 227 to 568 m and 6.9 to 8.3°C, respectively, appears to be critical habitat for *E*. *spinax* around Iceland. Although the surveys cover a large area of the sea around Iceland they do not cover all possible areas such as further down the continental shelf or along the Reykjanes ridge. Almost nothing is known of the reproductive cycle of *E*. *spinax* in other regions. Coelho and Erzini [10] hypothesized that mating might occur during the winter months in the northeastern Atlantic, and Capape *et al*. [50] suggested that the gestation period might be around one year, or even longer. Neither of these speculations offer insight into the discussion of critical habitat for the species. However, McMillan et al. [51] reported that large pregnant females were caught in the shallow waters in Langesund, Norway, at around 200–300 m depth. The authors suggested that the area might be a pupping ground or a pre-pupping ground, thus, it might possibly be a critical habitat. Yet, there is no indication from the survey abundance index that *E*. *spinax* numbers have changed over the past 11 years. Given the depth and location of its occurrence, continuation of the current survey time series would appear to be an excellent means for detecting any future shifts in the abundance and distribution of *E*. *spinax*. Extension of current fishing areas into the deeper waters around Iceland could potentially put *E*. *spinax* at risk.

## Supporting information

S1 File (XLSX)

S2 File (XLSX)
